# Deciphering the Binding of Salicylic Acid to *Arabidopsis thaliana* Chloroplastic GAPDH-A1

**DOI:** 10.3390/ijms21134678

**Published:** 2020-06-30

**Authors:** Igor Pokotylo, Denis Hellal, Tahar Bouceba, Miguel Hernandez-Martinez, Volodymyr Kravets, Luis Leitao, Christophe Espinasse, Isabelle Kleiner, Eric Ruelland

**Affiliations:** 1 IEES-Paris (UMR_7618)—Institut D’écologie et des Sciences de L’environnement de Paris; CNRS UMR 7583, Université Paris-Est Créteil, Sorbonne Université, F-94010 Paris, France; pokotylo@bpci.kiev.ua (I.P.); denishellal@gmail.com (D.H.); miangher@hotmail.com (M.H.-M.); luis.leitao@u-pec.fr (L.L.); christophe.espinasse@u-pec.fr (C.E.); 2 V.P. Kukhar Institute of Bioorganic Chemistry and Petrochemistry, National Academy of Sciences of Ukraine, 02094 Kyiv, Ukraine; kravets@bpci.kiev.ua; 3 Plateforme D’interactions Moléculaires, CNRS-FR3631; Institut de Biologie Paris Seine (IBPS), Sorbonne Université, Cedex 05, F-75252 Paris, France; tahar.bouceba@upmc.fr; 4 LISA (UMR 7583)—Laboratoire Interuniversitaire des Systèmes Atmosphériques (LISA), CNRS UMR 7583, Université Paris-Est Créteil, Université de Paris, Institut Pierre Simon Laplace (IPSL), 61 Avenue du Générale de Gaulle, F-94010 Créteil, France; kleiner@lisa.u-pec.fr

**Keywords:** salicylic acid, glyceraldehyde 3-phosphate dehydrogenase, molecular dynamics, molecular docking, protein ligand interaction, surface plasmon resonance, biacore

## Abstract

Salicylic acid (SA) has an essential role in the responses of plants to pathogens. SA initiates defence signalling via binding to proteins. NPR1 is a transcriptional *co*-activator and a key target of SA binding. Many other proteins have recently been shown to bind SA. Amongst these proteins are important enzymes of primary metabolism. This fact could stand behind SA’s ability to control energy fluxes in stressed plants. Nevertheless, only sparse information exists on the role and mechanisms of such binding. Glyceraldehyde 3-phosphate dehydrogenase (GAPDH) was previously demonstrated to bind SA both in human and plants. Here, we detail that the A1 isomer of chloroplastic glyceraldehyde 3-phosphate dehydrogenase (GAPA1) from *Arabidopsis thaliana* binds SA with a *K*_D_ of 16.7 nM, as shown in surface plasmon resonance experiments. Besides, we show that SA inhibits its GAPDH activity in *vitro*. To gain some insight into the underlying molecular interactions and binding mechanism, we combined in silico molecular docking experiments and molecular dynamics simulations on the free protein and protein–ligand complex. The molecular docking analysis yielded to the identification of two putative binding pockets for SA. A simulation in water of the complex between SA and the protein allowed us to determine that only one pocket—a surface cavity around Asn35—would efficiently bind SA in the presence of solvent. In silico mutagenesis and simulations of the ligand/protein complexes pointed to the importance of Asn35 and Arg81 in the binding of SA to GAPA1. The importance of this is further supported through experimental biochemical assays. Indeed, mutating GAPA1 Asn35 into Gly or Arg81 into Leu strongly diminished the ability of the enzyme to bind SA. The very same cavity is responsible for the NADP^+^ binding to GAPA1. More precisely, modelling suggests that SA binds to the very site where the pyrimidine group of the cofactor fits. NADH inhibited in a dose-response manner the binding of SA to GAPA1, validating our data.

## 1. Introduction

Salicylic acid (SA, 2-hydroxybenzoic acid) is an important phytohormone that controls defence reactions in plants challenged by pathogens [1,2]. SA is produced in the cells located at the site of the pathogen attack. In these cells, SA initiates downstream responses aiming at counteracting the attack. Besides, the methylation of SA into methyl salicylate (MeSA) by a SA methyl transferase increases its volatility and cellular permeability. MeSA could serve as a chemical signal travelling from the site of the infection to distant tissues. In these distant sites, MeSA is de-methylated back to SA, which then contributes to establishing a systemic immunity (systemic acquired resistance, SAR) [3,4]. SA is also a part of some abiotic stress reactions in plants, e.g., drought responses in oat [5] or salinity tolerance in *Arabidopsis thaliana* [6].

SA also has a role in animals, where salicylate-derived compounds are indeed being extensively used in medicine. Aspirin is acetylsalicylate. When administered orally, aspirin is rapidly converted into SA, which has a much longer half life as compared to its acetylated counterpart [7].

Signalling pathways initiated by SA in the cells are starting to be understood [8]. However, while SA has an essential role, either in the physiology of plants or both in human and animal health, only sparse information exists on the role of proteins that bind SA and on the mechanisms of these SA–protein interactions [2].

Interestingly, it seems that SA recognition and signalling does not implicate membrane receptors that would trigger cascades of protein kinases, leading to activation or the inhibition of target enzymes. This would represent a classical hormone pathway described in mammals and for plant brassinosteroids. On the contrary, it appears that SA directly binds to cytosolic proteins, potentially modulating their functions or enzymatic activity. SA binds to NPR1, which is an essential transcription co-regulator of the SA pathway. SA has a role in NPR1 monomerisation [9]. NPR1 monomers shuttle from the cytosol to the nucleus where they interact with TGA transcription factors, thus allowing the induction of SA-responsive genes in plants [8]. NPR1 is a critical protein component of the SA pathway and a great deal of SA sensitivity is lost in *npr1* plants [10]. Nevertheless, the binding of SA to NPR1 cannot explain the whole range of SA responses. First, some responses to SA seem to be NPR1-independent [11,12,13]. Recently, SA-induced attenuation of root growth in Arabidopsis was attributed to SA binding to A subunits of protein phosphatase 2 (PP2A) and the inhibition of its activity acting on PIN auxin efflux carriers. Such an effect of SA is well preserved in *npr1* plants [14]. Second, even within the NPR1-dependent pathway, SA binding to NPR1 does not explain it all. The monomerisation of NPR1 occurs through the reduction of disulphide bridges in the NPR1 homotetramer; binding to SA alone is not enough to monomerise NPR1 [9]. Moreover, NPR1 monomerisation could altogether be SA-independent. For instance, endoplasmic reticulum (ER) stress promoted NPR1 nuclear translocation, which could be only due to its monomerisation. During ER stress, SA is not accumulated but a redox potential of the cytosol is reduced [15]; this is the same effect observed following SA treatment [16]. This suggests that the role of SA in NPR1 monomerisation could be due to targeting protein regulators of redox status and not due to NPR1 binding *per se*. The cytosolic thioredoxins TRX-h3 and TRX-h5 were pulled down with the N-terminal part of NPR1 and were suggested to act in NPR1 monomerisation [17]. The regulation of their activity by SA is not confirmed. Finally, we have shown that SA rapidly activates phosphatidylinositol-4-kinases [18] and phospholipase D [19]. These activations are necessary for the triggering of the NPR1-dependent pathway [18,19]. Here again, SA response needs to occur through binding proteins that will trigger these lipid-signalling pathways upstream of NPR1. 

Proteins other than NPR1 that bind SA have been found. The first described salicylic acid binding protein (SABP1) was a catalase, which was identified in tobacco [20]. SABP2 is a methysalicylate esterase [21,22], and SABP3 is a beta carbonic anhydrase [23]. With the exception of SABP2, their exact roles in the SA signalling pathway were not documented. SA has also been shown to bind NPR1 paralogs [24,25]. Recently, by high-throughput biochemical screenings, nearly 100 soluble proteins were proposed to be able to bind SA in *Arabidopsis thaliana* plant cells [26]. Such a large number of proteins that potentially could bind SA is surprising. Indeed, for many hormones, only a limited number of proteins playing the role of receptors are usually found. Moreover, in this list of proteins, essential enzymes for the primary metabolism exist such as the small unit of the RUBISCO protein (AtRBCS1A, the key enzyme of the CO_2_ fixation during photosynthesis) and several isomers of *Arabidopsis thaliana* chloroplastic GAPDH that are important enzymes of photosynthesis: GAPA1 and GAPA2 [27]. Therefore, the understanding of the mechanism of the interaction between a protein and SA is more than ever a relevant biological question.

In humans, the mechanisms of salicylate or aspirin actions have also proven to be much more complex than initially suggested (having a role in cyclooxygenase inhibition). A number of protein targets of aspirin were identified [28]. SA also could bind human cytosolic GAPDH, acting in glycolysis [29]. Even though SA is not a hormone in animals, evolutionarily, this can be explained by SA uptake by animals from plant food sources.

Despite our knowledge of proteins that bind SA, we still know little about the mechanisms of such binding. For instance, in plants, it was shown that SA binds and inhibits catalase SABP1 [30] or thimet oligopeptidases TOP1 and TOP2 in vitro [31]. How this is happening on the molecular level is still unclear.

To elucidate the binding motifs and mechanism at an atomistic scale, combined methods of in silico simulations (molecular dynamics) with in vitro binding assays were applied. We started this study with a glyceraldehyde 3-phosphate dehydrogenase (GAPA1), which is a member of the evolutionary conserved glyceraldehyde 3-phosphate dehydrogenase (GAPDH) family. Our approach allowed us to identify a surface cavity, structured by Asn35, as the one allowing the binding of SA. Asn35 and Arg81 were shown to be important for such binding. Interestingly, this site is the very same site where the adenine part of the cofactor NAD(P) is accepted by GAPA1.

The approach we used can be applicable to other SA target proteins, either of plant or animal origin.

## 2. Results

### 2.1. GAPA1 Binds SA with High Affinity

**Figure 1:**
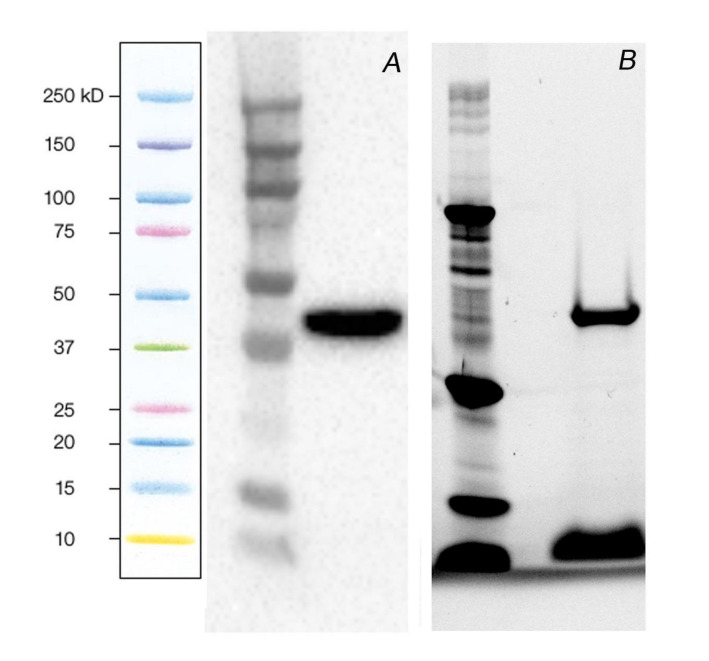
Production and purification of recombinant glyceraldehyde 3-phosphate dehydrogenase (GAPA1). In (**A**), a detection of GAPA1 with anti-6xHis antibodies in a soluble fraction of *E. coli* lysate via the peroxidase activity of secondary antibodies. In (**B**), a SDS-PAGE of GAPA1 purified from a soluble fraction of *E. coli* lysate using Ni-NTA resin. The protein band was visualised by stain-free imaging (Bio-Rad). Shading at the bottom of the track is due to the loading buffer pigments.

**Figure 2:**
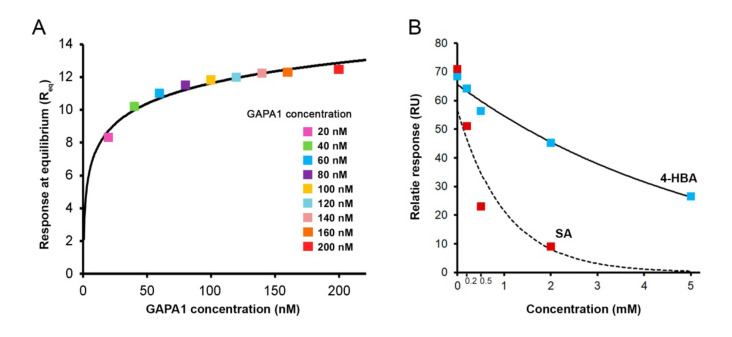
Assessment of GAPA1 binding to immobilised 3-aminoethyl salicylic acid (3-AESA) in surface plasmon resonance (SPR) assay. In (**A**), a range of GAPA1 concentrations were tested for their ability to bind immobilised 3-AESA. *K*_D_ of this interaction was calculated by fitting the binding profiles to a 1:1 Langmuir interaction model (Supplementary Figure S1A). Binding experiments were performed 3 times and led to a mean *K*_D_ value of 16.7 ± 5 nM. The Langmuir modelling allowed extracting the response at equilibrium (Req) values for each tested protein concentration. Req values were plotted against GAPA1 concentrations. In (**B**), GAPA1 ability to bind 3-AESA was assessed in the presence of an excess of SA or 4-HBA. For that, the concentration of GAPA1 was set at 200 nM. Samples were pre-incubated with an indicated concentration of SA on ice for 1 h prior to the binding test. Trend lines are generated for visualisation purposes.

GAPA1 was produced in *E. coli* and purified to more than 90% (Figure 1). Then, we studied its interaction with SA via surface plasmon resonance (SPR). In this technique, an aminoethyl derivative of SA—3-aminoethyl salicylic acid (3-AESA)—is immobilised on the sensor surface and serves as bait. A running buffer containing GAPA1 is flown over 3-AESA to detect binding in real time. GAPA1 bound 3-AESA in a concentration-dependent manner. Representative sensorgrams are available in Supplementary Figure S1A. These binding experiments were fitted to a 1:1 Langmuir model. From this model, an equilibrium dissociation constant (*K*_D_) could be calculated. The GAPA1-3–AESA interaction yielded to a mean *K*_D_ of 16.7 ± 5.7 nM in three independent assays. From the Langmuir modelling, responses at equilibrium (Req) could be extracted and plotted against the concentration of protein (Figure 2A). Note that no binding was observed when bovine serum albumin (BSA; used as a negative control) was flown over 3-AESA (Supplementary Figure S2).

Following the observation of a GAPA1 binding to 3-AESA, we then tested if this interaction was relevant in regard to the interaction with native SA. To do so, we have performed competition tests in the presence of an excess of SA. Relative responses were extracted just after the beginning of the dissociation phase of the sensorgrams (Supplementary Figure S1B,C). GAPA1 association with immobilised 3-AESA was inhibited by the presence of SA in a running buffer in a concentration-dependent manner (Figure 2B). This demonstrated that SA competes with 3-AESA for GAPA1 binding (*i.e.,* binding occurs at the same site/sites). The presence of 4-hydroxybenzoic acid (4-HBA), a biologically inactive structural isomer of SA, had led to a much weaker competition (Figure 2B).

### 2.2. SA Inhibits GAPA1 Catalytic Activity in Vitro

**Figure 3:**
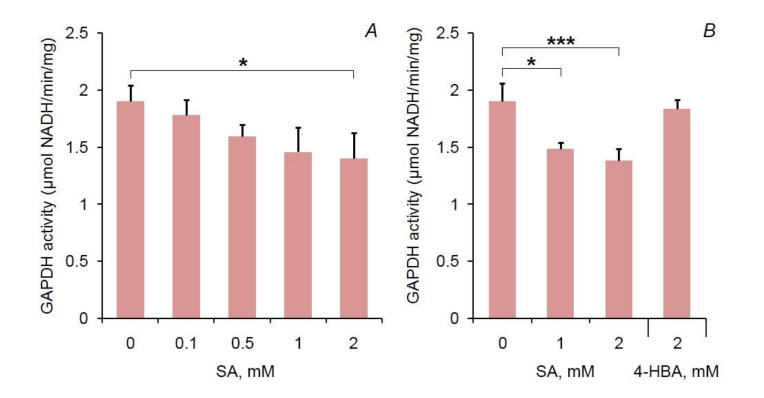
Glyceraldehyde 3-phosphate dehydrogenase (GAPDH) activity (NAD-dependent) of GAPA1 pre-incubated with SA or 4-hydroxybenzoic acid (4-HBA) on ice for 1 h. Graphs display averages of 3 replicates. Error bars show SD. Statistical differences were confirmed by ANOVA (panel **A**) and by a two-tailed Student’s t test (panel **B**): * (α = 0.05), *** (α = 0.001).

Then, we wanted to analyse the effect of SA on GAPDH enzymatic activity. GAPA1 was pre-incubated with SA on ice for 1 h prior to in vitro enzymatic assays. SA inhibited GAPA1 activity in a concentration-dependent manner (Figure 3A). Incubation with an equivalent concentration of 4-HBA did not lead to an inhibitory effect: at 2mM, SA produced 28% inhibition of GAPDH activity of GAPA1, while 2 mM of 4-HBA produced an inhibition of only 4% (statistically insignificant) (Figure 3B).

### 2.3. In Silico Modelling of SA Interaction with GAPA1

Our data strongly indicate that SA binds GAPA1, but these biological assays do not allow for a detailed understanding of the ongoing phenomena at the molecular level. Here, in silico techniques such as molecular dynamics simulations are the methods of choice to elucidate the underlying interaction mechanism at an atomistic scale. 

We extracted the structure of one GAPA1 monomer form the structure of the wild-type protein AtGAPDHA1 crystallised as a tetramer (PDB 3K2B; Supplementary Figure S3). Note that even though NADP is the preferred cofactor of the chloroplastic GAPA1, the authors crystallised it with NAD [32].

**Figure 4:**
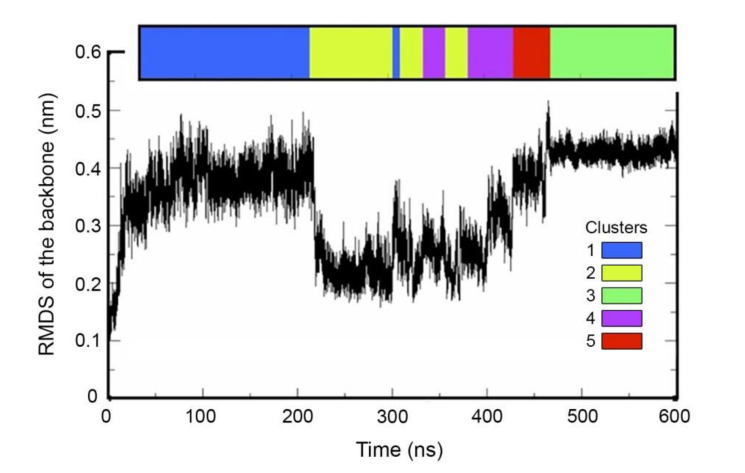
Relaxation of the protein structure in water. Root mean square deviations (RMSD) are shown for GAPA1 (GROMACS package v5.0.7). In RMSD, each structure from a trajectory is compared to a reference structure; here, the reference used is the structure of the protein after the pressure (NPT) equilibration step. The time frames corresponding to the 5 clusters are shown in colour. Clustering was performed for the simulation time 40 nsec to 600 nsec.

This initial structure was relaxed in water for 600 nsec using GROMACS package v5.0.7. The root mean square deviation (RMSD) of atomic positions, which is a measure of global backbone deviation, was calculated using the structure of the protein before the simulation started (i.e., after the pressure equilibration step) as the reference. The RMSD presents a quick increase that attains a plateau after 40 nsec; yet, a big decrease of about 0.2 nm is observed at 200 nsec of simulation; then, a sharp increase in RMSD occurs at around 410 nsec (Figure 4). A root mean square fluctuation (RMSF) was calculated between 0 and 75 nsec: the rapid change of the RMSF occurs in the vicinity of the residue Ala200 that corresponds to an unstructured loop (Supplementary Figure S4A). RMSF was also calculated for 190–250 nsec and 400–500 nsec simulation spans: the decrease in RMSD at 200 nsec and the subsequent increase are also due to fluctuation around Ala200 (Supplementary Figure S4A). 

Then, we performed a clustering of the structures obtained during the relaxation from 40 to 600 nsec with a sampling of 390 psec. We obtained 5 clusters (Figure 4). Clusters 1 to 3 comprise 73% of the simulation structures. Cluster 1 mostly corresponds to structures of the first plateau (40–190 nsec); cluster 3 mostly corresponds to structures of the 450–600 nsec plateau; cluster 2 is comprised of structures within the valley between 200 and 400 nsec of the simulation. For each cluster, an average structure was generated. By overlapping the average structures for clusters 1 to 3, we could visualise that the main difference between structures is due to the loop comprising Ala200 (Supplementary Figure S4B). Note that the second increase in RMSD leading to the second plateau (450–600 nsec) does not indicate reverting to structures similar to that of the first plateau (40–190 nsec). Indeed, when RMSD was calculated using the average structure 1 as the reference (Supplementary Figure S4C), we could see that structures in the second plateau (450–600 nsec) are in fact more distant to the structures of the first plateau (that are obviously the closest to average structure 1) than the structures of the valley (200–400 nsec).

#### 2.3.1. Preparation of the Ligand: Calculation of Atomic Charge of the Ligands

Molecular geometries of SA have been optimised using the *ab initio* method at MP2/6-311++G** level of theory using GAUSSIAN09 (https://gaussian.com). The SA PDB file used in our docking can be found as Supplementary File 1.

#### 2.3.2. Determination of SA-Binding Pockets

**Figure 5:**
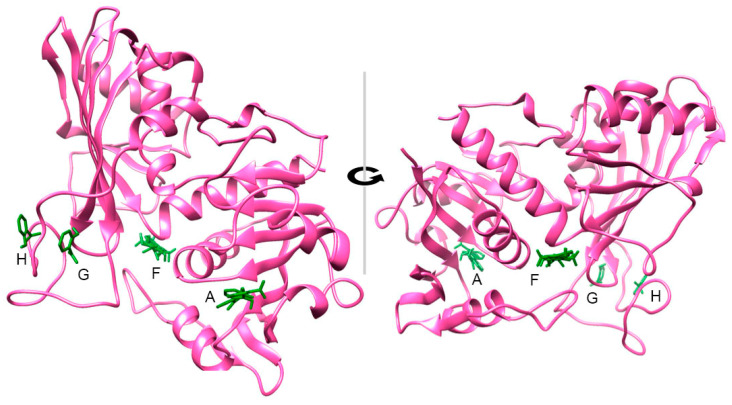
Docking positions of SA in GAPA1. The 10 best scores docking poses are shown in the average structure of cluster 3. SA is in green and the protein is in magenta. Docking was calculated by AUTODOCK VINA [33,34] implemented in PyMOL. Structures were visualised using CHIMERA [35].

AUTODOCK VINA [33,34] was used to perform the docking of SA in the average structures of clusters 1 to 3. Docking was performed without water molecules and with flexible side chains. As we did not know the binding pocket, a blind docking was performed enclosing the whole protein into the grid box. Standard default parameter settings were used. When considering these 10 best docking scores in the average structures of clusters 1 to 3, the SA was located in pockets labelled A to H (Table 1). Figure 5 illustrates those 10 best docking scores of SA in the average structure of cluster 3.

From Table 1, even though the scores are sometimes close to each other, it appears that two cavities, A and F, combine the highest scores and the main occurrence from the 3 clusters. For the average structure of cluster 1, the most represented cavity is cavity A, for which the highest scores are obtained. For the average structure of clusters 2 and 3, cavity F is the most represented and with the highest scores. In these two average structures, cavity A is nevertheless present amongst the 10 best scores, at positions 7 and 10 for average cluster 2 and positions 6, 8, and 9 for average cluster 3.

The GAPA1 monomer structure can be broadly defined as two sub-globuli with each globule being structured by a beta sheet (Supplementary Figure S3). Pocket A is within the amino terminal globuli; it is situated at the top of the beat sheet; it involves residues of the beta sheet such as Asn35 and Asn8, and residues in the loops between the end of the beta sheets and helices, such as Arg81 and Thr37. The upper side of cavity A is indeed capped by Arg81 and Thr37, while the bottom part of the cavity is comprised of Val102, Phe103, and Thr100. The inner part of the cavity is made of Asn35, Asp36, and Asn8 (Supplementary Figure S5A). Cavity F is at the interface of the two subparts of the peptide defined previously. It consists of Thr52 and Asp50 at one side and Ser290, Cys289, and Trp319 at the other side (Supplementary Figure S5B). When analysing the different docking possibilities of SA in cavity A (docking 1 to 4 and 6 in average structure of cluster 1), it appears that SA in cavity A is orientated in such a way that the negatively charged parts are towards the inside of the cavity, facing Asn35. Besides, the benzenic cycle is not necessary orientated the same way in all of the dockings. On the other hand, in pocket F (docking 1 to 6 and 8 in average structure of cluster 2), the benzenic cycle is always oriented parallel to the aromatic cycle of Trp319 (Supplementary Figure S6)). This suggests the importance of the pi–pi interactions in SA binding to cavity F. 

#### 2.3.3. Molecular Dynamics Simulations of the Complexes between SA and the Native GAPA1

**Figure 6:**
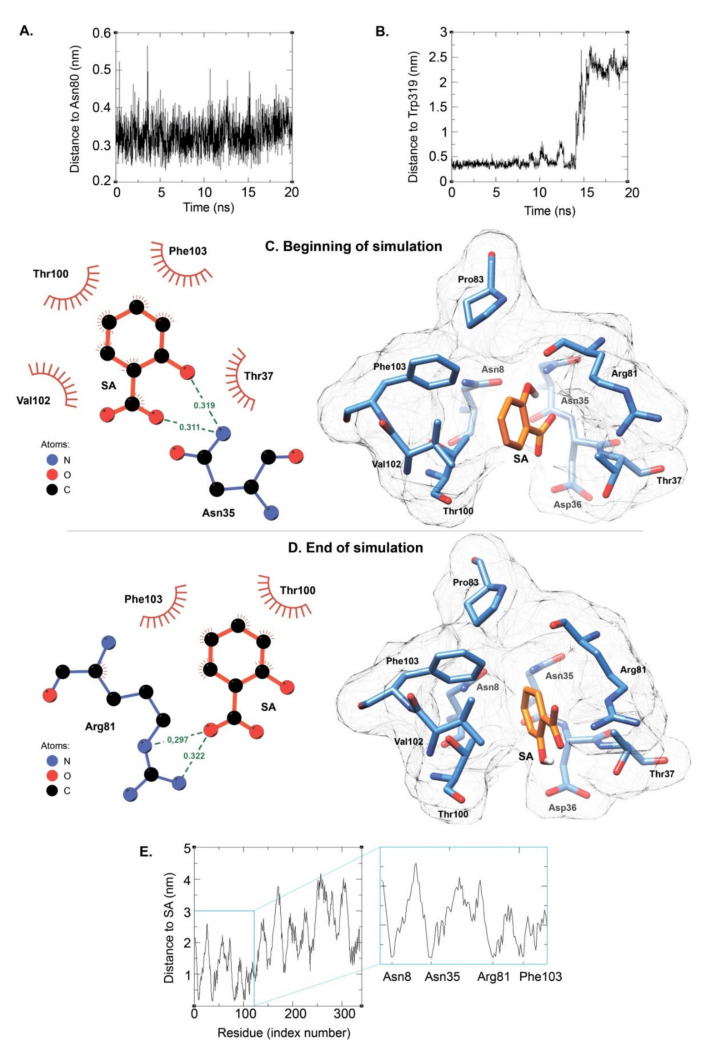
Molecular dynamic simulations of the complex between GAPA1 and SA. In (**A**), the distance between SA and Asn80 was calculated throughout a course of 20 nsec simulation; the starting docking corresponds to the best scored docking of SA in the average structure of cluster 1 (SA in pocket A). In (**B**), the distance between SA and Trp319 was calculated throughout a course of 20 nsec simulation; the starting docking corresponds to the best scored docking of SA in the average structure of cluster 3 (SA in pocket F). In (**C**), a position of SA is shown immediately after the temperature and pressure equilibrations using GROMACS package v5.0.7. In (**D**), a position of SA in the pocket is shown after a 20 nsec simulation in water. The starting docking used to generate panels (**C**) and (**D**) corresponds to the best scored docking of SA to the pocket A in the average structure of cluster 1. Structures were visualised using CHIMERA [35] and LigPlot^+^ [36]. H-bonds and distances (in nm) are shown as dashed green lines. Red spoked arcs represent protein residues making nonbonded contacts with the ligand. In (**E**), the distances between SA and each residue of GAPA1 were calculated during the 10 last nanoseconds of the simulation of SA docked to pocket A of the average structure of cluster 1.

For both cavities A and F, we performed five 20 ns simulations of the protein/SA complex in water with GROMACS package v5.0.7 to make sure that the complex is stable. We calculated the distance of SA to Asn80 and to Trp319 for pocket A and pocket F, respectively. We considered that SA is no longer bound to the pocket if the distance to the reference residue exceeded 1 nm in the course of the 20 nsec simulation. Interestingly, out of 5 simulations, SA drifted away from pocket F in 4 of these simulations. On the contrary, SA remained in the pocket A for this period of time in 4 out of 5 20 nsec simulations. In these simulations, the minimal distance from SA to Asn80 fluctuated in the range of 0.23 nm to 0.91 nm. Representative examples of stay/leave behaviour of SA, as shown on distance graphs, can be seen at Figure 6A,B. In pocket A, at the beginning of the simulation, SA was positioned with the -OH and -COOH groups towards the inside of the pocket (Figure 6C), pointing towards Asn35, thus producing H bonds. Throughout the simulation, SA made a full spin so that these groups are positioned towards the exit of the pocket, interacting with Arg81 (Figure 6D). H-bonds and hydrophobic interactions were calculated using LigPlot^+^ v2.2 software. Throughout the 10 last nanoseconds of the complex simulation, the four residues calculated to be the closest to SA are Asn8, Asn35, Arg81, and Phe103 (Figure 6E). 

#### 2.3.4. Molecular Dynamics Simulations of the Complexes between SA and Mutated AtGAPHA1

**Figure 7:**
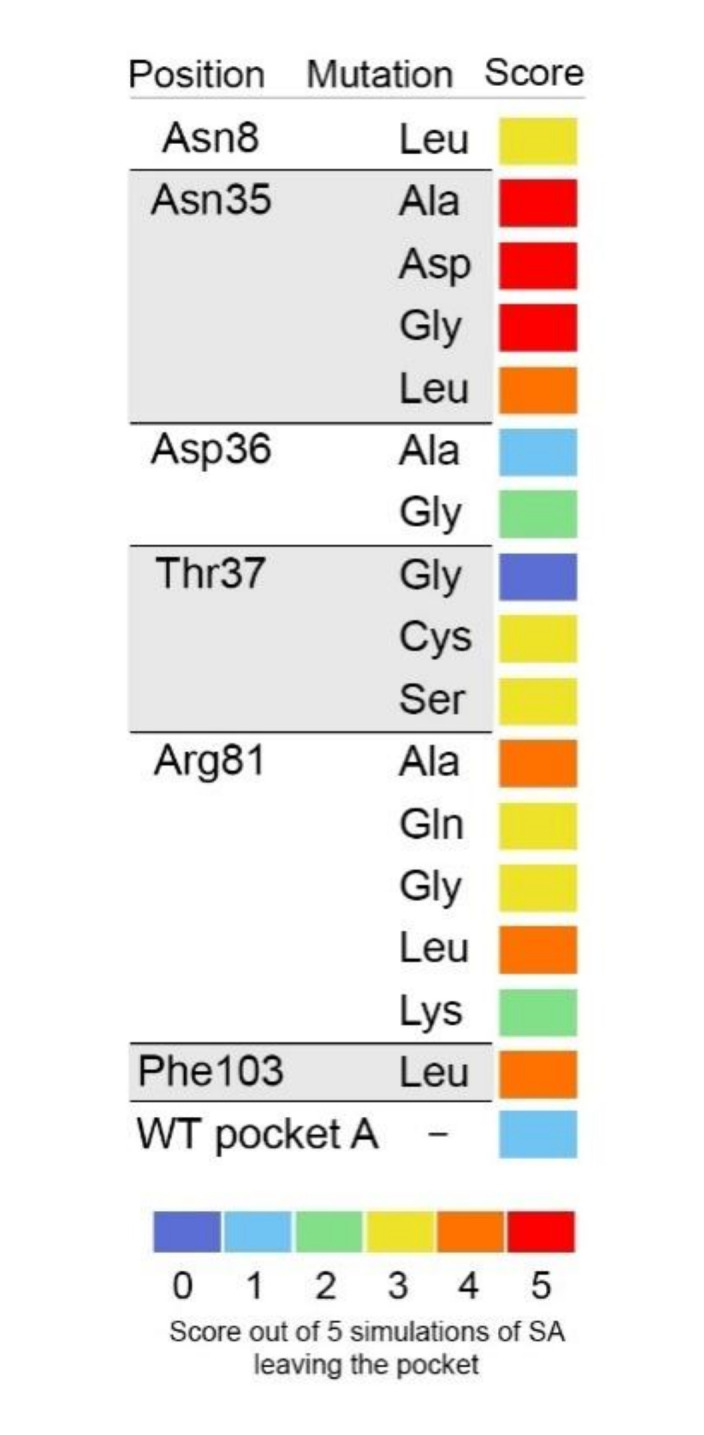
Twenty nsec simulations of the complexes between SA and mutated GAPA1. SA was docked in pocket A before simulation started. This docking of SA corresponds to the docking scored 1 in Table 1 for the average structure of cluster 1. The in silico mutations are performed on the average structure of cluster 1. Simulations were run 5 times from 5 independent energy minimisation steps. Criteria for assuming ligand leaving the pocket: distance to Asn80 greater than 1 nm.

In *silico* mutagenesis were performed on selected residues of pocket A. Similarly to native proteins (see above), 5 simulations were performed for each mutation. SA was considered to leave the cavity if the distance to Asn80 was higher than 1 nm (Figure 7).

Arginine is a residue with a positively charged lateral chain. When Arg81 was mutated into Ala or Leu, i.e., residues with hydrophobic side chains, SA left the cavity in 4 out of the 5 simulations in either case. When Arg81 was mutated into Gln, a residue with a polar but uncharged lateral chain, SA left the cavity in 3 out of the 5 simulations. It is also the case when Arg81 is mutated into Gly, for which the lateral chain is a H. This shows the importance of the positive charged lateral chain of Arg81 in the interaction with SA. When Arg81 was mutated into Lys, another positively charged residue, SA left the cavity in only 2 out of 5 simulations. We could observe that in the Agr81Lys mutant, the SA position in the cavity is also stabilised by producing an H-bond with Thr37 (Supplementary Figure S7A). This is also the case for some simulations in Arg81Ala and Arg81Gly (Supplementary Figure S7B,C). Therefore, Thr37, a residue that is not in close contact with SA in native pocket A (Figure 7E), could have some role in the docking of SA, especially in the case of Arg81 substitution. In the Thr37Ser and Thr37Cys mutants, SA left the pocket in 3 out of 5 simulations. Yet, when Thr37 is mutated into Gly for which the lateral chain is H, SA always stayed in the pocket. In this mutant, SA produced an H-bond with Arg81, similarly to the wild-type (WT) protein (Supplementary Figure S7D). Clearly, the role of Thr37 in the docking of SA is lesser to that of Arg81.

Asn35 was shown to be one of the closest residues to SA (Figure 6E). Asparagine has a polar yet uncharged lateral chain. Asn35 was mutated into Ala and Leu, which are residues with apolar lateral chains, or into Asp, a polar residue with a negatively charged chain, or Gly, for which the lateral chain is H. In all cases, this led to SA leaving the pocket in at least 4 out of the 5 simulations. As explained above, during the course of simulation in the WT protein, SA goes from a position where its -OH and -COOH groups are directed towards Asn35 (towards the inside of the pocket) to a position where they are positioned towards Arg81, i.e., to the outside of the pocket (Figure 6C,D). Hence, the importance of Asn35 is not to directly interact with SA, but more likely to structure the pocket. Indeed, Asn35 is shown to establish H-bonds with the backbone chain between Arg81 and Asn80 and Asn80 and Ser79; Asn35 is also calculated to establish a bond with Asn8 (data not shown). Asn35 is clearly at the interface of different peptide sections that constitute the pocket.

When Asn8 was mutated into Leu, SA left the pocket in 3 out of 5 simulations. Here again, SA could be stabilised by producing an H-bond with Thr37 (Supplementary Figure S7E). Phe103 defined one part of the entrance to the pocket. When Phe103 was mutated into Leu, SA left the pocket in 4 out of 5 simulations. This suggests that pi–pi interactions between the aromatic rings of Phe and SA could be of importance for retaining the latter in the cavity.

The residue 36 of GAPA1 is an aspartate, which is a polar negatively charged residue. Since it is near Asn35, we tested its possible role. When Asp36 was mutated into Ala or Gly, SA stayed in the pocket 4 and 3 times out of 5, respectively (Figure 7).

#### 2.3.5. SA is Docked to the NADPH Binding Pocket

**Figure 8:**
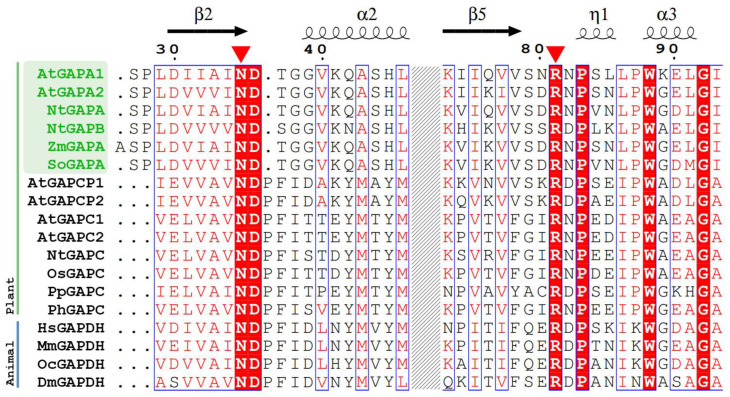
Alignment of different GAPDHs. Regions around Asn35 and Arg81 (marked with red triangles) of AtGAPA1 are shown. Plant NADP-dependent chloroplastic GAPDHs that are involved in photosynthesis are highlighted in green. AtGAPCP2 and AtGAPCP1 are NAD-dependent enzymes that function in chloroplasts. Animal and other plant GAPDHs are the cytosolic ones, while NAD-dependent ones are involved in glycolysis. Alignment was performed by ClustalX2. Conservation score is depicted using standard colour scheme of ESPript [37]. Residues are shown on red background if strictly conserved. At, *Arabidopsis thaliana*; Nt, *Nicotiana tabacum*; Zm, *Zea mays*; So, *Spinacia oleracea*; Os, *Oryza sativa*; Pp, *Physcomitrella patens;* Ph, *Petunia hybrid*; Hs, *Homo sapiens*; Mm, *Mus musculus*; Oc, *Oryctolagus cuniculus;* Dm, *Drosophila melanogaster*.

**Figure 9:**
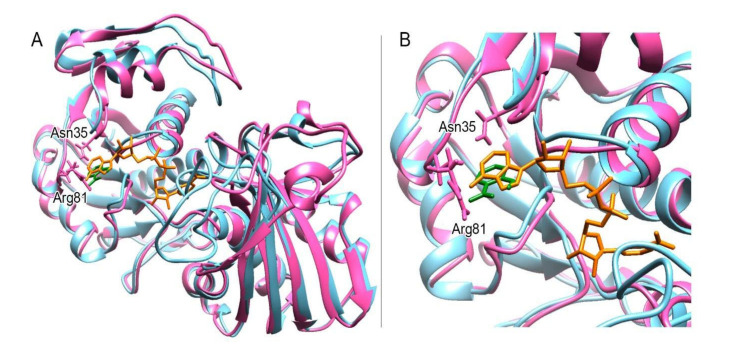
The SA binding pocket is the pocket where the adenine part of the NAD(P) cofactor fits. Structure in magenta: the protein structure in complex with SA after a 20 nsec simulation (such as in Figure 6A); structure in blue: the crystallographic structure corresponding to PDB 3K2B; orange: NAD; green: SA. (**A**) Overlapping between 3K2B and our simulation. (**B**) Close-up of A. Structures were visualised using CHIMERA [35].

We wanted to know whether the residues we identified are conserved amongst GAPDHs. We retrieved GAPDH sequences from animals and plants; for plants ones, we considered NAD-dependent ones and NADP-dependent ones as well (Figure 8). We could see that Asn35 and Arg81 are conserved in the assessed proteins. To what functions do they correspond? To understand that, we overlapped the crystal structure of GAPDH-A1 (PDB 3K2B) with the structure obtained at 20 nsec of the SA/WT protein complex simulation. In their structural work [32], the authors crystallised GAPDHA1 in the presence of NAD even though NADP is the preferred cofactor of this enzyme. Interestingly, SA overlaps with the adenine group of the NAD cofactor (Figure 9).

### 2.4. Testing the Ability of Point-Mutated GAPA1 to Bind SA

Based on in silico data, a cavity around Asn35 and involving Arg81 (cavity A) was suggested to be important for SA binding in GAPA1. Pre-selected residues of this cavity (Arg81 and Asn35) were validated by biochemical assays. Point-mutated GAPA1 proteins were produced and compared for their ability to bind SA in a similar SPR technique used for WT GAPA1. This allowed us to test if in vitro assays are in agreement with our theoretical predictions.

**Figure 10:**
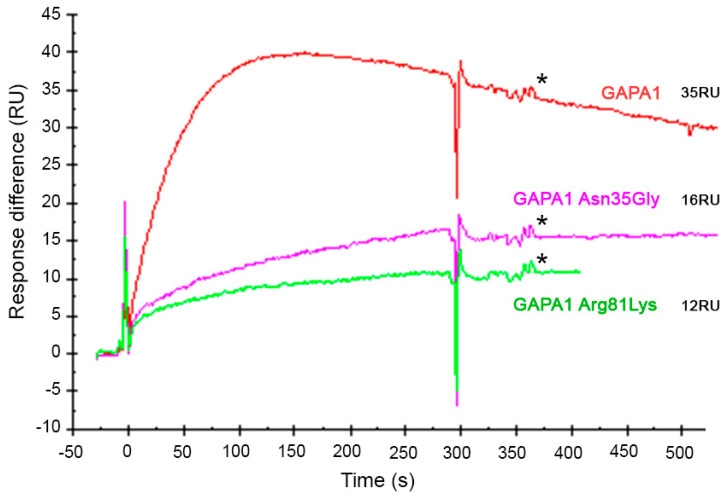
Comparison of the abilities of wild-type (WT) GAPA1 (red line) and point-mutated GAPA1 Asn35Gly (pink line) and GAPA1 Arg81Lys (green line) to bind immobilised 3-AESA in SPR assays. All proteins were injected in 50 nM concentration. In these sensorgrams, the signal from the mock-coupled surface was subtracted. The response values of report points (*) after the beginning of the dissociation phase were extracted. The corresponding relative responses are indicated in Resonance Units (RU). The report points are indicated by *.

In GAPA1 Arg81Leu, the ability to bind 3-AESA (as shown by relative responses) was diminished by 55%. Whereas in GAPA Asn35Gly, it was diminished by 66% (Figure 10), suggesting that these amino acid residues contribute to SA binding to GAPA1. No complete loss of ability to bind 3-AESA was observed in these mutated proteins. This could be due to the fact that (unlike simulations obtained in *silico*), singular point mutation does not completely disrupt interaction with SA. Due to the problems with protein stability, we could not produce an Arg81Leu–Asn35Gly double-mutated GAPA1. 

In the docking calculations, we had also identified a second potential SA binding site (cavity F) implicating Trp319. This cavity had a much lower score of retaining SA in molecular dynamic simulations. Indeed, we found out that the ability of GAPA1 Trp319Leu to bind 3-AESA was not affected (Supplementary Figure S8).

### 2.5. Ability of GAPA1 to Bind SA is Inhibited by NADH

**Figure 11:**
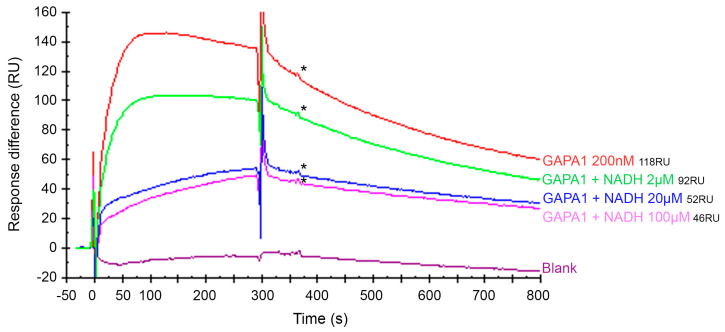
The ability of WT GAPA1 to bind immobilised 3-AESA is inhibited in the presence of NADH. GAPA1 concentration was set at 200 nM concentration. The response values of report points (*) after the beginning of the dissociation phase were extracted. The corresponding relative responses are indicated in Resonance Units (RU). The report points are indicated by *. In these sensorgrams, the signal from the mock-coupled surface was subtracted.

We have obtained preliminary data indicating that SA binding to GAPA1 could overlap with NAD(P) cofactor (Figure 9). In order to confirm such an observation, we have tested the ability of GAPA1 to bind immobilised 3-AESA in the presence of NADH. We used NADH as a competing molecule, since GAPA1 was crystallised with it [32]. Indeed, we have found that the pre-incubation of GAPA1 on ice with NADH reduced the level of GAPA1 association with immobilised 3-AESA (Figure 11). The competition was observed as soon as 2 µM NADH.

## 3. Materials and Methods

### 3.1. Protein Production and Purification

GAPA1 coding sequence (excluding the signal peptide) was amplified from a plasmid pda3105 obtained from The RIKEN BioResource Research Center and cloned into pET100/D-TOPO vector (Invitrogen, Waltham, MA, USA)). Insert orientation and sequence was verified by sequencing (Eurofins, Ebersberg, Germany). Recombinant GAPA1 was produced in *E. coli* BL21 (DE3) as a fusion protein with 6xHis-tag and purified by affinity purification with Ni-NTA resin (Thermo Scientific, Waltham, MA, USA). Proteins were buffer-exchanged to phosphate-buffered saline (PBS) by dialysis, concentrated by centrifugation columns (Amicon, Darmstadt, Germany), aliquoted, and stored in 50% (*v*/*v*) glycerol at −80 °C for further use.

### 3.2. Protein Mutation

Point mutations to GAPA1 were introduced by replicating the vector using primers that introduce desired substitutions to protein coding sequence (Q5 Site-Directed Mutagenesis Kit, New England Biolabs, Ipswich, MA, USA). Mutations were verified by sequencing (Eurofins, Ebersberg, Germany). 

### 3.3. GAPDH Activity Assay

The GAPDH activity of GAPA1 was measured in vitro via colorimetric assay according to the protocol provided by the manufacturer (ab204732, Abcam, Cambridge, UK).

### 3.4. Computational Methods: Starting Structures

For *Arabidopsis thaliana* AtGAPDHA1, we used the crystallographic structure of the photosynthetic homotetrameric (A_4_) glyceraldehyde-3-phosphate dehydrogenase A1 (PDB 3K2B) [32]. In silico mutagenesis was performed in CHIMERA [35], using the rotamer tool.

### 3.5. Ab initio Calculations for the Ligand

Molecular geometries of the considered Ligand (SA) have been optimised using the ab initio method at the MP2/6-311++G** level of theory using GAUSSIAN09. Harmonic vibrational frequencies of the different stationary points have been calculated at the same level. 

### 3.6. Molecular Dynamics Simulations

Protons were added using CHIMERA [35]. The GROMACS package v5.0.7 [38] was used to run our molecular dynamics simulations. The AMBER99SB-ILDN [39] force field was used to provide molecular mechanics parameters to our proteins. The proteins were put in a cubic cell (“box”), the border of which is at least 1 nm from the protein, and we solvate it with TIP3P [40] explicit water molecules. Counterions (Na^+^) were added to obtain a neutral system and took the place of water molecules. Then, the protein prepared was first submitted to a quick energy minimisation to ensure that the system has no steric clashes or inappropriate geometry and to avoid unnecessary distortion of the protein when the molecular dynamics simulation is started. The step size of the steepest descent minimisation was 50,000 and minimisation stopped when the maximum force was <1000.0 kJ/mol/nm. Next, we performed two equilibration runs. Bonds will be constrained to enable 2 fs time steps. The first equilibration was performed to reach the target temperature of 300 K in the NVT ensemble (constant Number of particles, Volume, and Temperature) using the modified V-rescale Berendsen thermostat [41] with 100 ps relaxation time. The second equilibration, that is equilibration of pressure, was conducted in the NPT ensemble (wherein the Number of particles, Pressure, and Temperature are all constant) using the Parrinello–Rahman barostat [42] set at 1 bar as reference pressure. The equilibration time was also set at 100 ps relaxation time. 

Once our protein was well-equilibrated at the desired temperature and pressure, we release the position restraints and run production MD for data collection. The wild protein (AtGAPDHA1) was subjected to 600 ns simulation with 2 fs time steps. After the simulation was done, we centred the protein in the box and calculate the RMSD (root mean square deviations) and the root mean square fluctuation (RMSF) tools implemented in GROMACS package v5.0.7.

Structures of the protein obtained during simulation were clustered using the Ensemble Cluster tool implemented in the CHIMERA visualisation software [35]. This tools clusters members of a conformational ensemble (i.e., the different structures generated through the simulation) and determines cluster representatives. It is a reimplementation of the method described previously [43].

Docking was performed using AUTODOCK VINA [33,34] implemented as a plug-in in the PYMOL visualisation software. Docking box dimensions were specified so that the protein was surrounded by this box. A flexible docking procedure was applied.

### 3.7. Simulation of the Complex between SA and the Protein

The force field parameters for the ligand were calculated using the ACPYPE software (AnteChamber PYthon Parser interface) [44]. The charge for SA was *n* = −1. The protein/ligand complex was simulated during at least 20 nsec to check its stability. The complex was built and the unit cell was defined in GROMACS package v5.0.7. After solvation and the addition of ions, the energy of the system was minimised. To do so, first, a position restraint topology was generated for the ligand. Second, the ligand was coupled to the protein for appropriate control of temperature using the make_ndx command of GROMACS package v5.0.7. Equilibration steps and molecular dynamics were done as previously described with energygrps = Protein LIG.

The protein/SA complexes with the highest docking scores were subjected to 20 to 30 ns simulations. 

### 3.8. Analysis of the MD Simulations

Visualisation of protein structures with or without ligands were visualised by Chimera [35]. For visualisation reasons, the solvent molecules (water) were not considered.

### 3.9. Surface Plasmon Resonance Analysis

The surface plasmon resonance (SPR) biosensor experiments were performed on a Biacore 3000 instrument (GE Healthcare) of the Platform of Molecular Interactions of the Institute of Biology Paris Seine (IBPS, Sorbonne University). The CM5 sensor chip was used. 3-AESA (called the ligand) was immobilised through primary amino groups to the carboxymethyl dextran matrix of a CM5 sensor chip. 3-AESA (10 mM) diluted in immobilisation buffer (0.1 M Borate) was injected at a flow rate of 10 μL/min for 30 min. The immobilisation was followed by an injection of 70 μL of ethanolamine hydrochloride (1 M) pH 8.5, at a flow rate of 10 μL/min, to saturate the free activated sites of the matrix. A reference surface without ligand was prepared using the same procedure. Buffer HBS-EP (10 mM HEPES, pH 7.4, 150 mM NaCl, 3 mM EDTA, 0.005% surfactant P20) was used as the running buffer and for diluting all the injected molecules. The variations of SPR signal as a function of time (sensorgram) were obtained by passing various concentrations of the injected protein or small molecule (called the analyte) over the ligand surface at a flow rate of 5 μL/min, with an association phase of 5 min and a dissociation phase of 8 min. The surface of the sensor was regenerated with an injection of 25 mM NaOH at a flow rate of 10 μL/min and 30 s contact time. Identical injections over blank surfaces executed in parallel (giving a value of 0 RU) were subtracted from all experiments. The kinetics were evaluated using the BIAevaluation software, version 4.1 (GE Healthcare, Chicago, IL, USA). The data were processed by fitting the binding profiles to a 1:1 Langmuir interaction model with a subsequent extrapolation of response at equilibrium values (Req). The quality of the fit was assessed by the statistical Chi2 value provided by the software (Chi2 values < 10 were considered as acceptable). The fitting of each dataset yielded rates for association (k_a_) and dissociation (k_d_), from which the dissociation constant *K*_D_ was calculated (*K*_D_ = k_d_/k_a_). The k_on_, k_off_, and *K*_D_ from 3 experiments were used to calculate the mean values of these variables. 

## 4. Discussion and Conclusions

### 4.1. SA Binds Multiple Proteins but No Common Pattern is to Be Expected

SA’s ability to bind multiple protein targets and to affect their enzymatic activities is an intriguing phenomenon concerning not only plant physiology but also therapeutic applications of SA derivatives (aspirin). Despite opulent data on the identification of new proteins that bind SA, we still know little about mechanisms of such binding. No conserved SA-binding motif is known, and interaction could occur by various scenarios. For an NRP1, SA binding implicates Cys521/529 and requires the presence of copper ions [9]. No co-crystallisation data of NPR1-SA are yet available. Cys521/529 are not conserved in NPR1 orthologs that nevertheless efficiently bind SA [24]. Not to mention other classes of SABPs. NtSABP2 was co-crystallised with SA and Ser81, Ala13 and His238 residues were producing hydrogen bonds with a carboxylic group of SA [22]. This implies that the characterisation of SA–protein interaction each time will be a bespoke task. Our goal was to propose a new workflow strategy that combines in silico tools with subsequent biochemical validation to study SA–protein interactions at the molecular level.

### 4.2. GAPA1 is a High-Affinity SABP

In this study, we investigated a mode of interaction with SA of a GAPA1 from *Arabidopsis thaliana*, which was previously reported to be an SABP [27]. GAPA1 is a chloroplastic GAPDH that primarily acts in photosynthesis in the Besnon–Calvin cycle.

GAPA1 bound 3-AESA with high affinity *K*_D_ = 16.7 nM in SPR analysis. SPR is a powerful approach that provides high-quality data describing the kinetics of protein–ligand interactions as shown for SA and a number of proteins [45,46]. 

We concluded the interaction to be biologically relevant, since binding competition with SA was much greater than that with 4-HBA, despite the fact that these two molecules are very close from the chemical point of view.

The GAPA1 level of affinity to SA is relatively high when compared to other characterised SA–protein interactions. For example, SABP1 bound SA with a *K*_D_ of 14 μM [47], SABP2 bound SA with *K*_D_ = 90 nM [21], whereas NPR1 bound SA with *K*_D_ = 140 nM [9]. High Mobility Group Box 3 (HMGB3) is an example of an SABP with higher affinity to SA (*K*_D_ = 1.5 nM) [46] compared to GAPA1. It has to be mentioned that not the same methods were used to assess the binding of these proteins, so some discrepancy is to be expected.

### 4.3. SA Binds GAPA1 via Arg81 and Asn35

Knowing that SA binds GAPA1, it was important for us to establish which amino acid residues are involved. To do so, we used a protocol that first establishes these residues in molecular dynamics simulations. The first step is protein relaxation in water to obtain representative structures of different clusters; then, each representative (“an average structure”) is used for docking calculation. By testing this protocol, we could well replicate the position of SA binding in NtSABP2 where this position is known based on co-crystallisation data [22]. Then, docking poses were assessed by simulating the complex between SA and the protein. In the case of GAPA1, such simulations yielded the identification of a surface pocket that could bind SA. The entrance to this pocket is capped by Arg81 and Thr37. Arg81 was shown to be the residue to which SA establishes a bond in the native protein. This was confirmed by using mutated proteins in the simulations. The importance of Asn35 in the binding was also shown; we suggest that the role of Asn35 is in the structuring of the cavity. By establishing in silico which AA residues could be involved in SA binding, we tested if this is corroborated by in vitro assays. The ability of GAPA1 N35G and R81L to bind 3-AESA in SPR assays (the same protocol as the one to applied WT GAPA1) was indeed significantly reduced.

In this way, our computation approach that use molecular dynamics tools proved to be an efficient strategy for providing primary data as of possible sites of SA–protein interaction. At the same time, we strongly believe that such computational data should always be corroborated by in vitro tests before establishing interaction metrics. In optimistic scenarios, such an approach could greatly speed up finding a place where proteins interact with SA or other ligands for that matter. 

### 4.4. SA Inhibits GAPDH Activity of GAPA1. Does the Same Concern Human GAPDH?

In our tests, we have observed that SA inhibits the GAPDH activity of GAPA1 *in vitro*. Inhibition occurred at rather high SA concentrations (2 mM) that are not expected to occur *in planta* (basal SA is around 1 µg/g FW [48]. Still, the biological grounds of this effect are validated by the fact that 4-HBA in the same concentration tested (2 mM) had not induced inhibition. GAPA1 is localised in chloroplasts–organelles that harbour the key pathway for the SA biosynthesis–chorismate pathway. So, one can expect that local SA concentration will be higher in chloroplasts before SA is distributed to cytosol via transporters [49] or passive diffusion. Endogenous SA concentrations are also multifold-increased following SAR [50] or in SA-overaccumulating mutants [51].

Corroborating the fact that SA could indeed be an inhibitor of GAPA1 activity is its localisation in binding complex with the protein. SA is bound exactly at the site of NADP docking the enzyme (Figure 9). In GapB—a GAPDH from spinach—Arg77 (corresponding to Arg81 in GAPA1) was shown to interact with negatively charged 2′-phosphate of NADP [52]. Besides, in GapB R77A, the thioredoxin-mediated redox regulation was abolished and enzyme activity was diminished in vitro [53]. Thus, SA could potentially impede NADP docking to GAPDHs and prevent enzyme catalytic functions/regulation. NADH and SA indeed compete for GAPA1 binding (Figure 11).

The fact that small molecules (such as SA) go into the binding site of a cofactor is not uncommon in enzymology. Cofactor binding sites are targeted by many drugs [54]. For instance, the NAD/NADH cofactor binding site of parasitic S-adenosyl-L-homocysteine hydrolase is targeted for the design of anti-parasitic drugs [55]. The NADH-binding site of the trypanosomal GAPDH was suggested to be a site of choice to develop selective inhibitors against trypanosomes [56]. There are similar concerns for other cofactors. It is well known that the majority of kinase inhibitors, including dasatinib and PP1, bind at the ATP-binding site [57].

By observing the aligned sequences of GAPDH from different plant species as well as from animals, we have observed that Arg81 and Asn35 are conserved in all GAPDHs (Figure 8). Considering the fact that other plant GAPDH (GAPA2, GAPC1, and GAPC2), as well as human GAPDH also bind SA, it is tempting to speculate that binding occurs at the same position as in GAPA1. This data would be of great importance for analyzing the therapeutic application of SA. In our studies, we have observed that Thr37 could play some role in SA binding (Figure 7). In NAD-dependent GAPDH (either in plants or in animals), position 37 is proline (Figure 8). However, this should not impede potential interactions with SA, since a change of Thr37 to Pro has not led to SA leaving the binding pocket in our simulations (data not shown).

### 4.5. Role of SA Binding and Inhibiting GAPA1 in Plant Stress Responses

However, what could be a biological outcome for SA binding GAPA1 in plants? Chloroplastic GAPDHs are key enzymes of photosynthesis, so SA inhibiting GAPA1 could represent a mechanism for SA to take control of primary metabolism. An observed effect of SA on GAPA1 could similarly have connections to its potential non-canonical activities. For many GAPDH enzymes (mainly glycolytic), numerous so-called moonlighting activities, that are alternative, additional functions in respect to their originally assigned ones, were described [58,59]. GAPCp1 is a GAPDH from wheat that directly interacts with the cytochrome b6-f complex iron sulfite subunit (Cytb6f) (in yeast two-hybrid and bimolecular complementation assays). The latter protein has a role in ROS scavenging and could be implicated in drought responses [60].

Moreover, a number of GAPDH enzymes have roles in direct association to pathogen responses. Cytosolic GAPC-1 in *A. thaliana* could directly associate with a negative RNA strand of the tomato bushy stunt virus (TBSV), which was a prerequisite for its asymmetric replication. Binding to SA abolished such activities of GAPC-1 [27]. In *Nicotiana benthamiana*, a chloroplast-localised GAPDH-A interacted with Red clover necrotic mosaic virus (RCNMV) movement protein (MP) [61]. The silencing of NbGAPDH-A resulted in the inhibition of virus multiplication in leaves and prevented MP targeting to the viral replication complex (used by RNA viruses for genome replication) located at a cortical endoplasmic reticulum [61]. A role of SA in this reaction has not been established.

However, no roles outside housekeeping functions were so far reported for GAPA1 from *Arabidopsis thaliana*.

GAPA1-knockout resulted in a slight increase of Arabidopsis resistance to *Pseudomonas syringae* pv. *tomato inoculation* [58], whereas the expression of GAPA1 is significantly reduced following 3 h post-infiltration with 5 μM flg22, which is an elicitor of plant defences [58]. These data provide evidence that GAPA1 could be a negative regulator of immunity. Whether this is due to GAPDH activity or another function of GAPA1, and the role of SA in these reactions, have yet to be established.

## Figures and Tables

**Table 1:** Docking of salicylic acid (SA) in the average structures of clusters 1, 2 and 3. Docking was performed with AUTODOCK VINA [33,34] implemented in PyMOL. Spacing was set at 0.375 for grid definition.

Average Structure Cluster 1			Average Structure Cluster 2			Average Structure Cluster 3		
Rank ^a^	Score ^b^	Pocket ^c^	Rank ^a^	Score ^b^	Pocket ^c^	Rank ^a^	Score ^b^	Pocket ^c^
1	–5.9	A	1	–6	F	1	–6.3	F
2	–5.6	A	2	–5.9	F	2	–6.2	F
3	–5.5	A	3	–5.8	F	3	–5.7	G
4	–5.4	A	4	–5.7	F	4	–5.5	F
5	–5.3	B	5	–5.5	F	5	–5.5	F
6	–5.2	A	6	–5.4	F	6	–5.4	A
7	–5.2	C	7	–5.3	A	7	–5.4	F
8	–5.2	B	8	–5.3	F	8	–5.3	A
9	–4.9	D	9	–5.2	C	9	–5.3	A
10	–4.8	E	10	–5.2	A	10	–5.2	H

For the different protein structures, the docking poses are sorted from 1 to 10 (a) according to the calculated scores by AUTODOC VINA as a sum of intermolecular and intramolecular contributions (b). Each docking pose was visualised in the protein structure, thus allowing the naming of the pockets (c). Some docking poses are indeed in the same pockets.

## Supplementary Materials

Supplementary Materials can be found at https://www.mdpi.com/1422-0067/21/13/4678/s1.
