# Correction: Pokotylo, I., et al. Deciphering the Binding of Salicylic Acid to *Arabidopsis thaliana* Chloroplastic GAPDH-A1. *Int. J. Mol. Sci.* 2020, *21*, 4678

**DOI:** 10.3390/ijms21207435

**Published:** 2020-10-09

**Authors:** Igor Pokotylo, Denis Hellal, Tahar Bouceba, Miguel Hernandez-Martinez, Volodymyr Kravets, Luis Leitao, Christophe Espinasse, Isabelle Kleiner, Eric Ruelland

**Affiliations:** 1 IEES-Paris (UMR_7618)—Institut D’écologie et des Sciences de L’environnement de Paris, CNRS UMR 7583, Université Paris-Est Créteil, Sorbonne Université, F-94010 Paris, France; pokotylo@bpci.kiev.ua (I.P.); denishellal@gmail.com (D.H.); miangher2010@gmail.com (M.H.-M.); luis.leitao@u-pec.fr (L.L.); christophe.espinasse@u-pec.fr (C.E.); 2 V.P. Kukhar Institute of Bioorganic Chemistry and Petrochemistry, National Academy of Sciences of Ukraine, Murmanska 1, 02094 Kyiv, Ukraine; kravets@bpci.kiev.ua; 3 Plateforme D’interactions Moléculaires, CNRS-FR3631, Institut de Biologie Paris Seine (IBPS), Sorbonne Université, Cedex 05, F-75252 Paris, France; tahar.bouceba@upmc.fr; 4 LISA (UMR 7583)—Laboratoire Interuniversitaire des Systèmes Atmosphériques (LISA), CNRS UMR 7583, Université Paris-Est Créteil, Université de Paris, Institut Pierre Simon Laplace (IPSL), 61 Avenue du Générale de Gaulle, F-94010 Créteil, France; kleiner@lisa.u-pec.fr

The authors wish to make the following correction to their published paper [1]. 

In Figure 10, the response difference curve for the GAPA1 Arg81Leu protein was mislabelled as GAPA1 Arg81Lys. In Figure 10’s caption, “GAPA1 Arg81Lys” should be “GAPA1 Arg81Leu”. The corrected Figure 10 is shown below (Figure 1)

We apologize for any inconvenience brought to the readers.

## Figures and Tables

**Figure 1:**
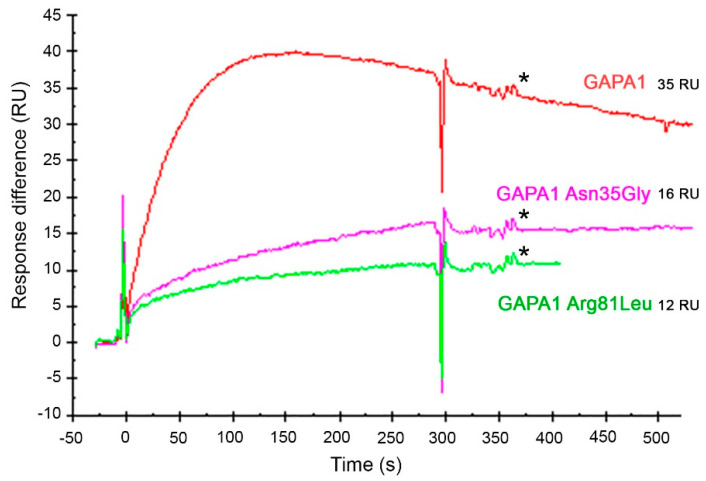
Comparison of the abilities of wild-type (WT) GAPA1 (red line), point-mutated GAPA1 Asn35Gly (pink line) and GAPA1 Arg81Leu (green line) to bind immobilised 3-AESA in SPR assays. All proteins were injected at 50 nM concentrations. In these sensorgrams, the signal from the mock-coupled surface was subtracted. The response values of the report points (*) after the beginning of the dissociation phase were extracted. The corresponding relative responses are indicated in Resonance Units (RU). The report points are indicated by *.
