# Supplementary figures and images for: Deciphering the Binding of Salicylic Acid to Arabidopsis thaliana Chloroplastic GAPDH-A1
Source: Int J Mol Sci. 2020 Jun 30;21(13):4678. doi: 10.3390/ijms21134678 (PMC7370300; doi:10.3390/ijms21134678)

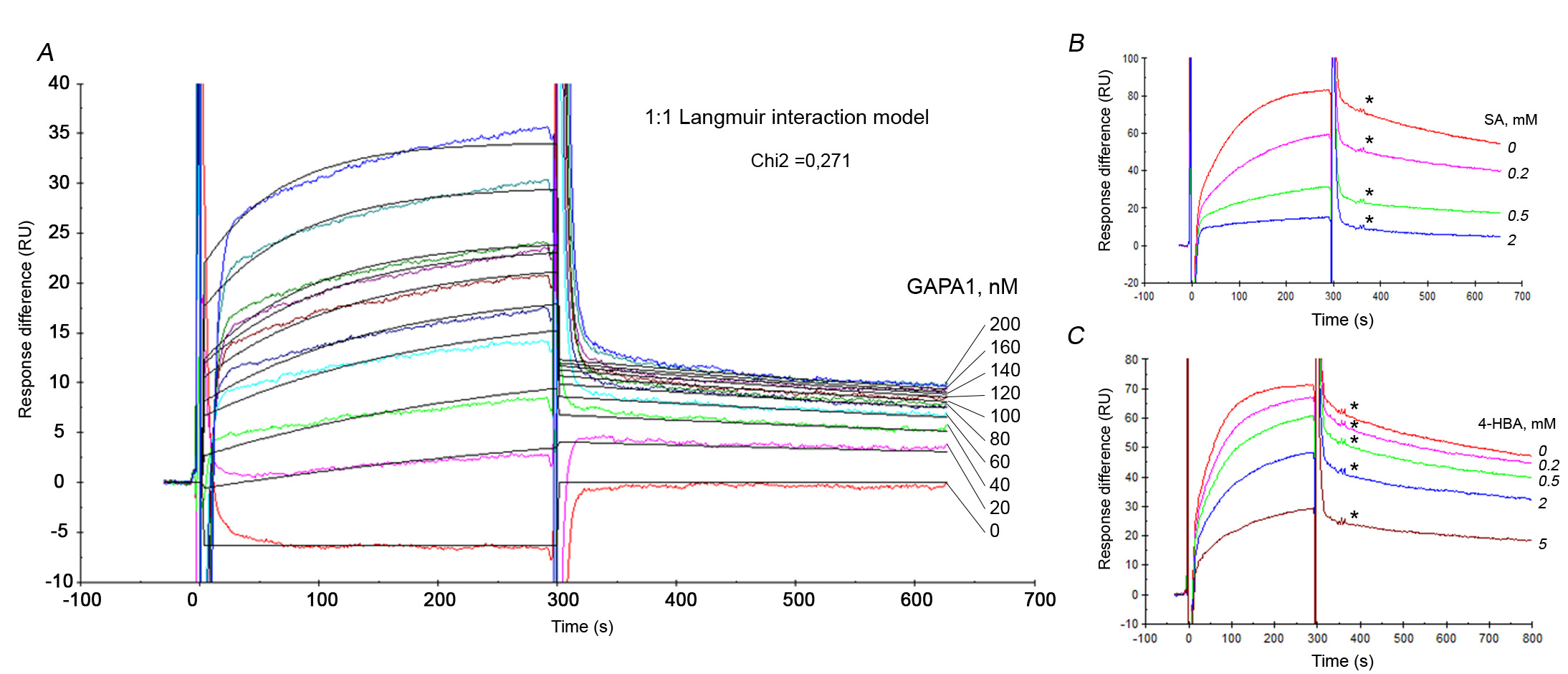

Supplement: Supplementary file 1 [file ijms-21-04678-s001.zip › Supplementary Figure S1.jpg]

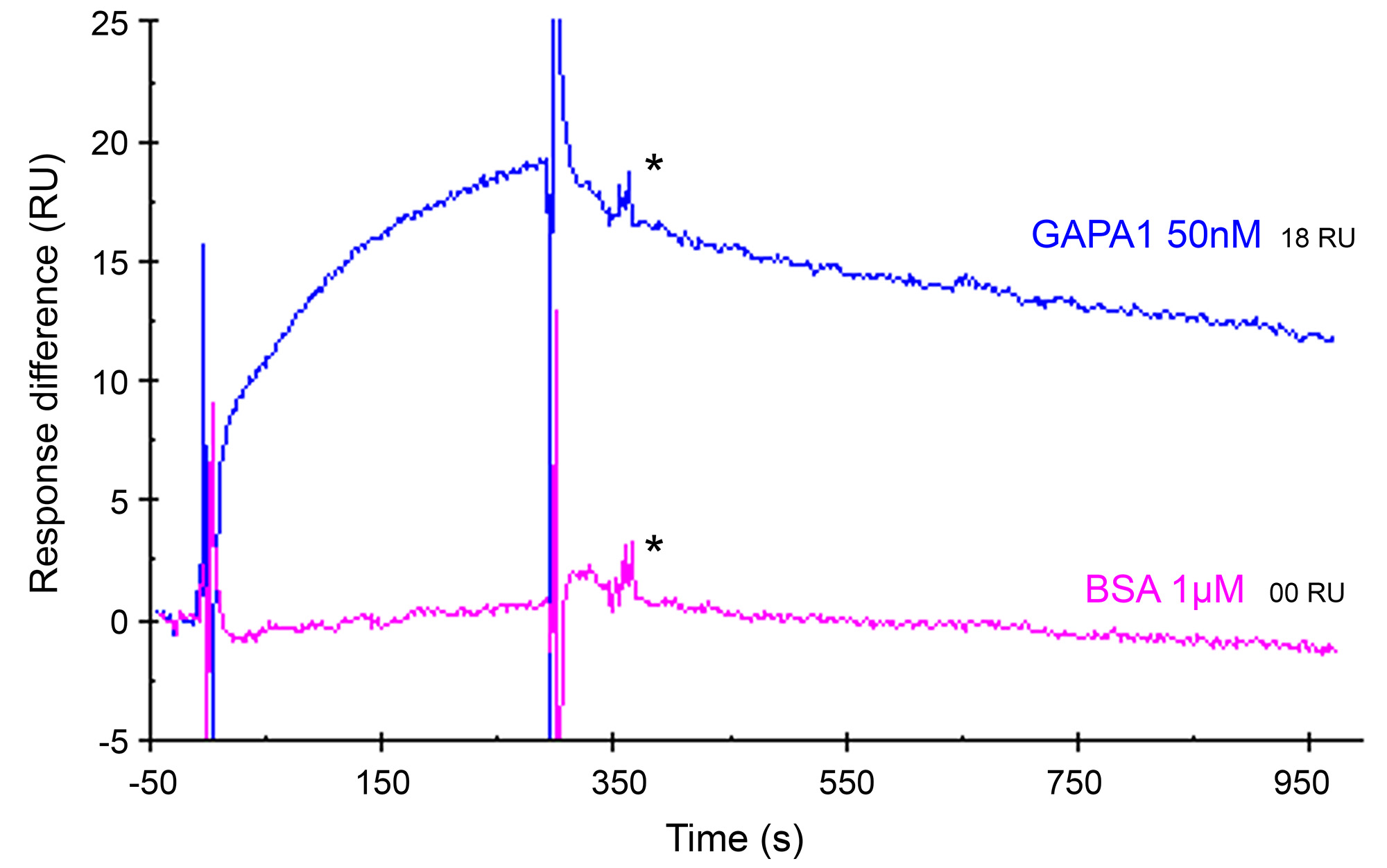

Supplement: Supplementary file 1 [file ijms-21-04678-s001.zip › Supplementary Figure S2.jpg]

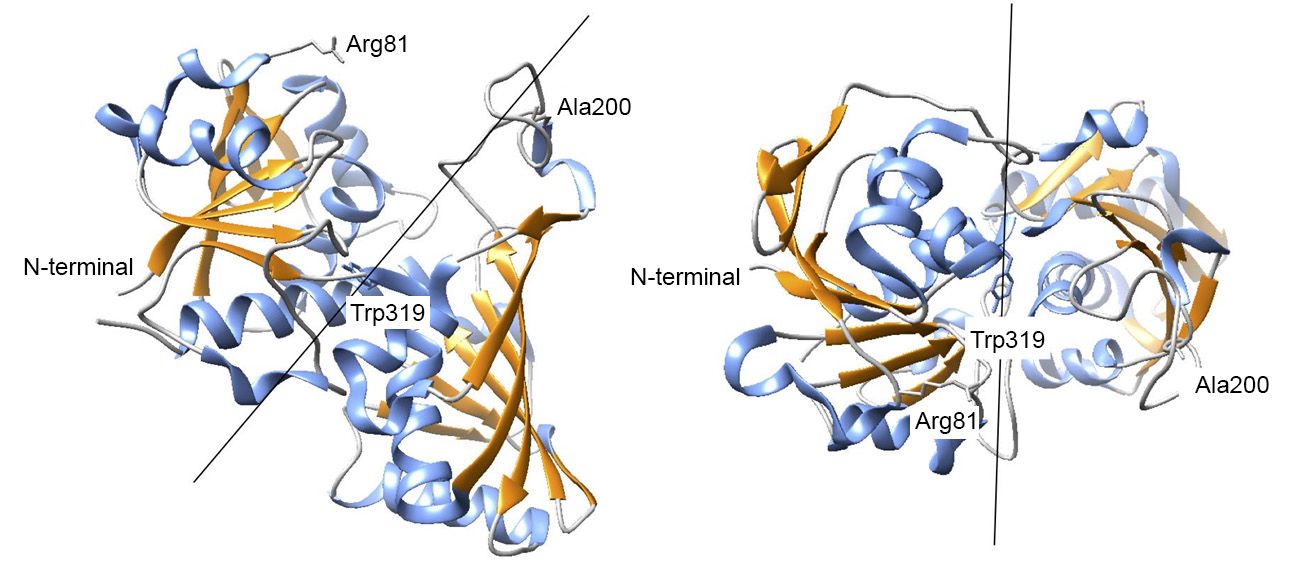

Supplement: Supplementary file 1 [file ijms-21-04678-s001.zip › Supplementary Figure S3.jpg]

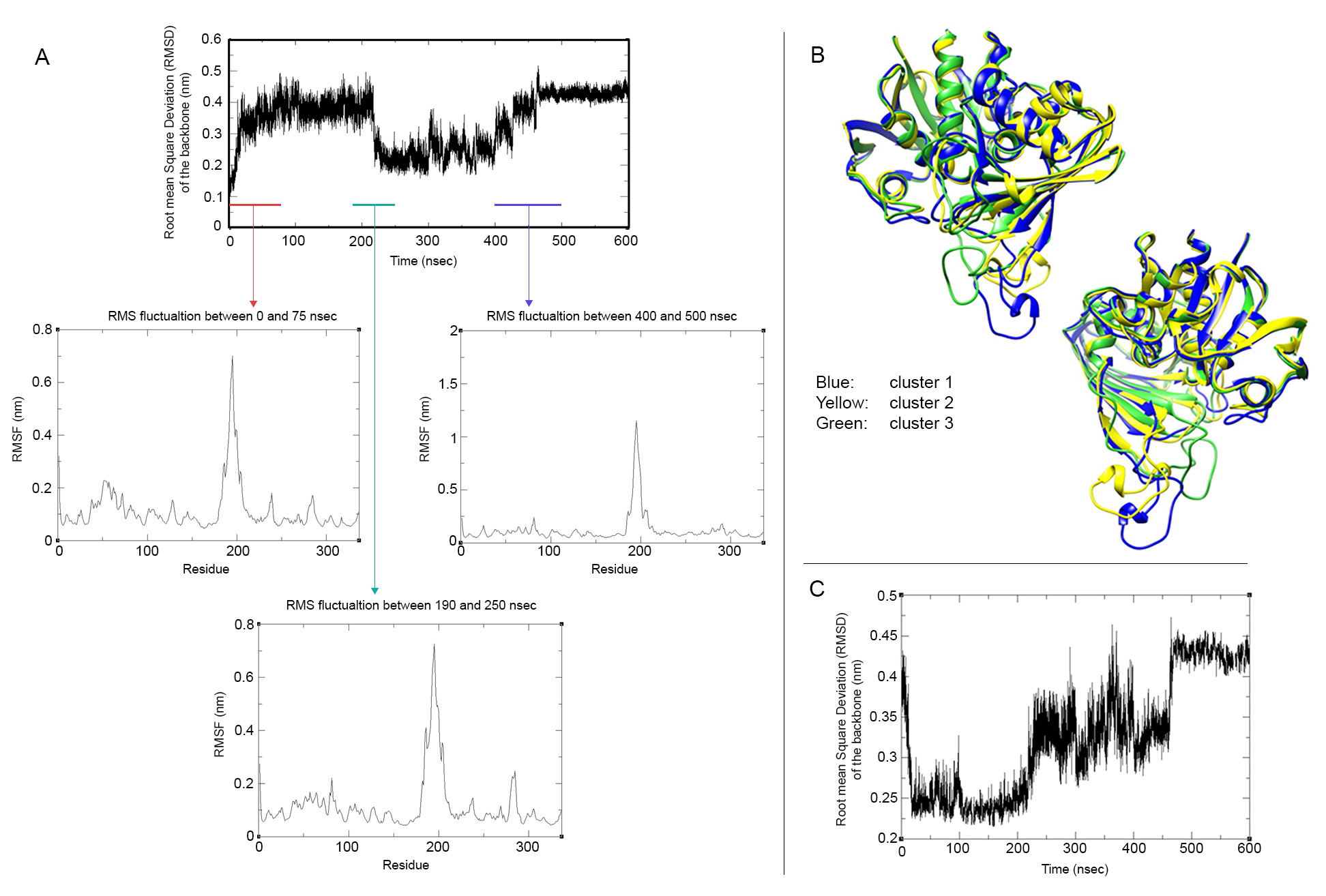

Supplement: Supplementary file 1 [file ijms-21-04678-s001.zip › Supplementary Figure S4.jpg]

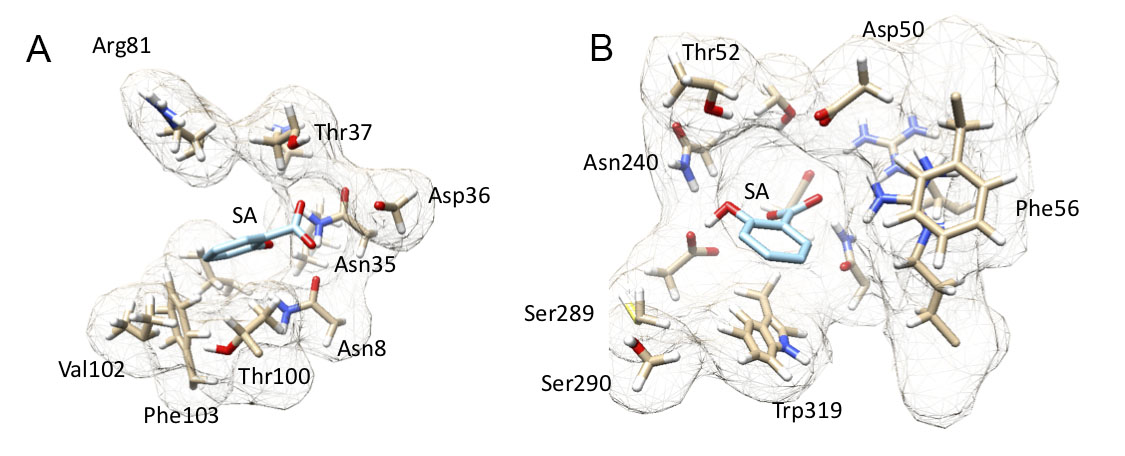

Supplement: Supplementary file 1 [file ijms-21-04678-s001.zip › Supplementary Figure S5.jpg]

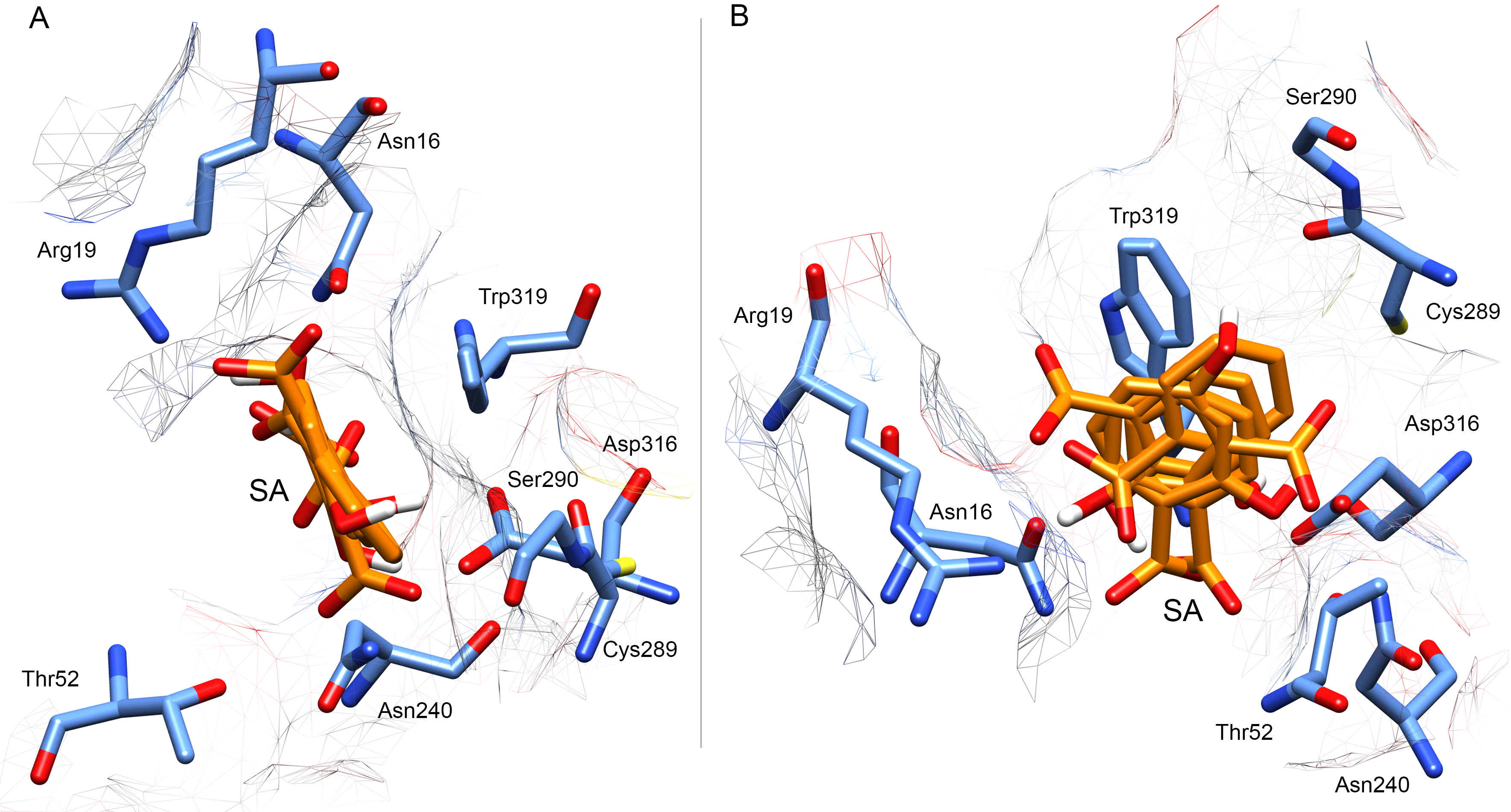

Supplement: Supplementary file 1 [file ijms-21-04678-s001.zip › Supplementary Figure S6.jpg]

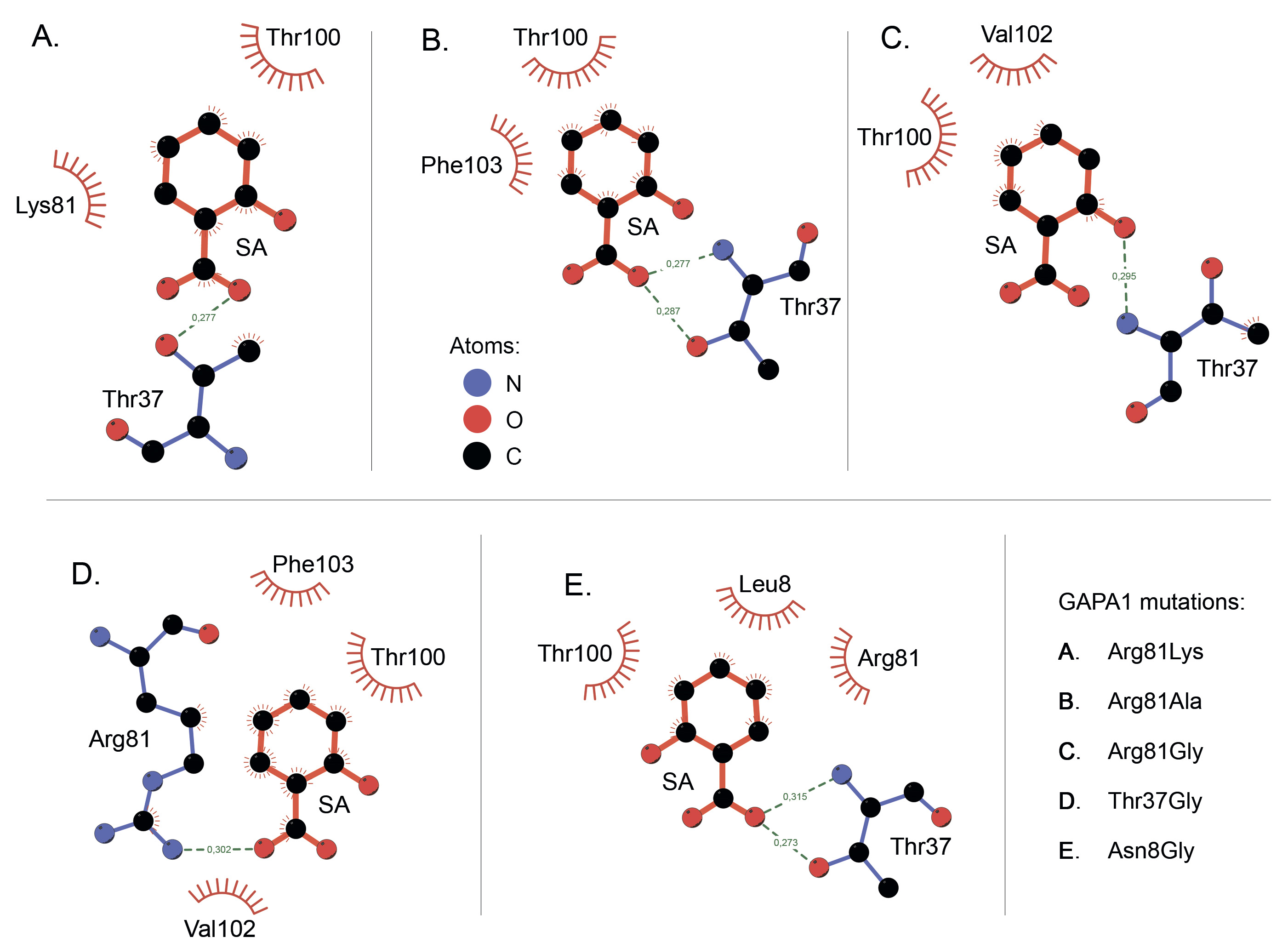

Supplement: Supplementary file 1 [file ijms-21-04678-s001.zip › Supplementary Figure S7.jpg]

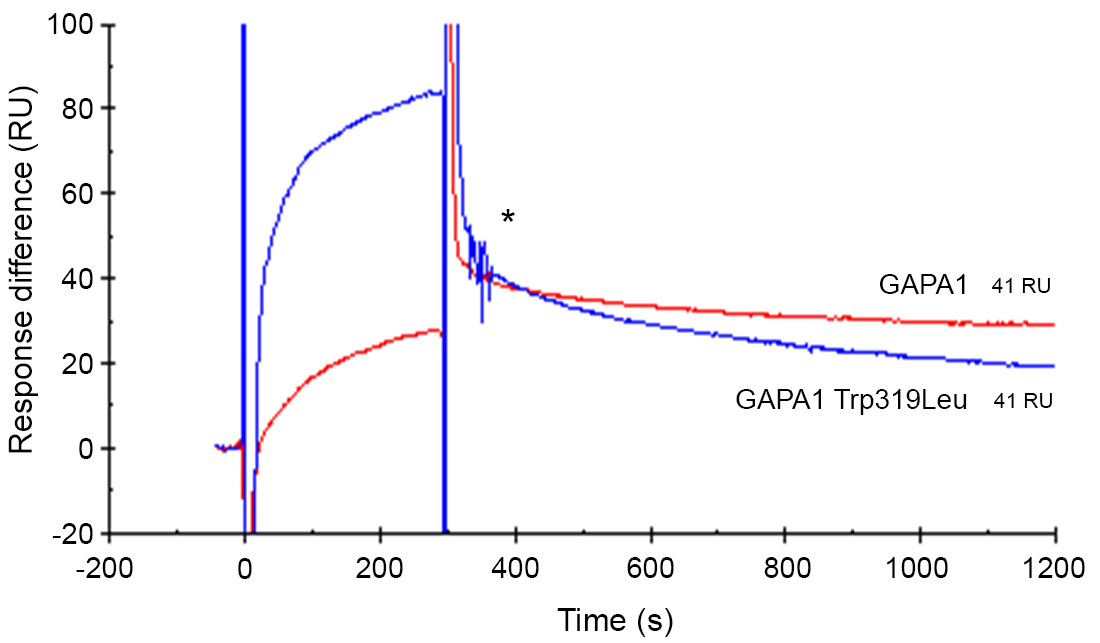

Supplement: Supplementary file 1 [file ijms-21-04678-s001.zip › Supplementary Figure S8.jpg]
